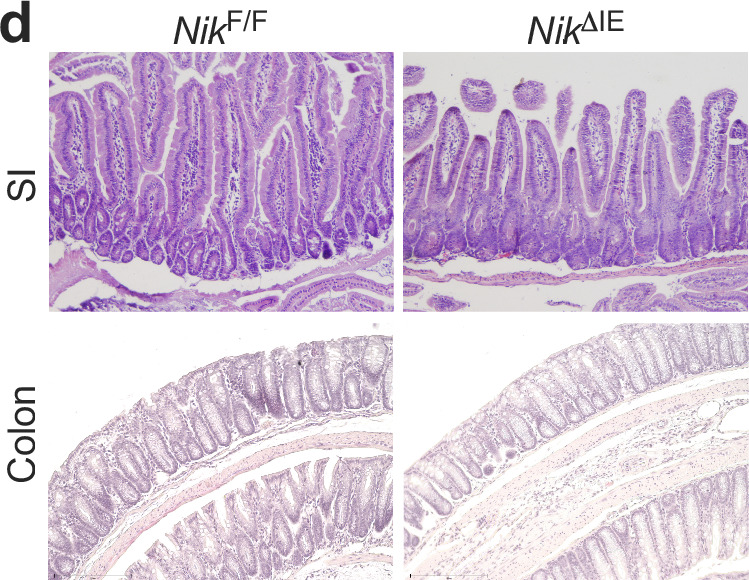# Author Correction: Intestinal non-canonical NFκB signaling shapes the local and systemic immune response

**DOI:** 10.1038/s41467-025-61581-9

**Published:** 2025-07-08

**Authors:** Sadeesh K. Ramakrishnan, Huabing Zhang, Xiaoya Ma, Inkyung Jung, Andrew J. Schwartz, Daniel Triner, Samantha N. Devenport, Nupur K. Das, Xiang Xue, Melody Y. Zeng, Yinling Hu, Richard M. Mortensen, Joel K. Greenson, Marilia Cascalho, Christiane E. Wobus, Justin A. Colacino, Gabriel Nunez, Liangyou Rui, Yatrik M. Shah

**Affiliations:** 1 Department of Medicine, University of Pittsburgh, Pittsburgh, PA 15261 USA; 2 Department of Molecular & Integrative Physiology, University of Michigan, Michigan, MI 48109 USA; 3 Department of Pathology, University of Michigan, Ann Arbor, MI 48109 USA; 4 Internal Medicine, University of Michigan, Ann Arbor, MI 48109 USA; 5 Cancer and Inflammation Program, Center for Cancer Research, National Cancer Institute, National Institutes of Health, Frederick, MD 21702 USA; 6 Transplantation Biology, University of Michigan, Ann Arbor, MI 48109 USA; 7 Department of Surgery, University of Michigan, Ann Arbor, MI 48109 USA; 8 Department of Microbiology and Immunology, University of Michigan, Ann Arbor, MI 48109 USA; 9 Department of Environmental Health Sciences, University of Michigan, Ann Arbor, MI 48109 USA; 10 Department of Nutritional Sciences, University of Michigan, Ann Arbor, MI 48109 USA; 11 Comprehensive Cancer Center, University of Michigan, Ann Arbor, MI 48109 USA

Correction to: *Nature Communications* 10.1038/s41467-019-08581-8, published online 8 February 2019

In the version of the article initially published, there was an error in Fig. 1d in which the lower left (NikF/F, Colon) image was inadvertently used again in the lower right (NikdIE, Colon) image. This error has been corrected by replacing both images with new regions from the samples of the correct genotypes. The corrected panel is shown as Fig. 1 below. This notice serves to amend the error. No update has been made to the original paper.

Fig. 1 | Corrected Fig. 1d