# Supplementary material for: Intestinal non-canonical NFκB signaling shapes the local and systemic immune response
Source: Nat Commun. 2019 Feb 8;10:660. doi: 10.1038/s41467-019-08581-8 (PMC6368617; doi:10.1038/s41467-019-08581-8)
Supplement: Supplementary file 1 — Supplementary Information [file 41467_2019_8581_MOESM1_ESM.pdf]

Supplementary Information

Intestinal non-canonical NFκB signaling shapes the local and systemic immune response.

Ramakrishnan et al.

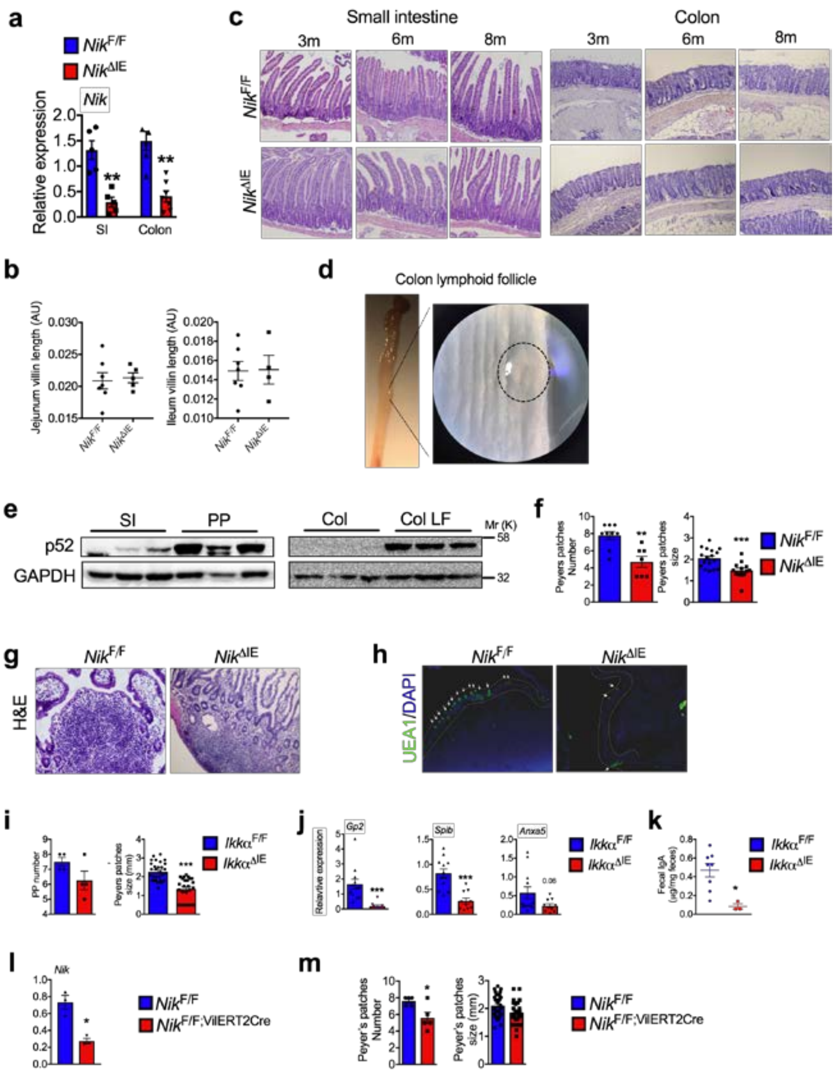

Supplementary Fig. 1

Epithelial NIK-IKKα signaling is essential for M-cell maintenance. **a** Expression of NIK assessed by qPCR in the small intestine (SI) and colon of 6-week old $Nik^{F/F}$ and $Nik^{\Delta IE}$ mice. **b** Intestinal villi length (Jejunum, left; ileum, right) assessed in 6-week old $Nik^{F/F}$ and $Nik^{\Delta IE}$ mice. **c** H&E analysis of small intestine and colon of $Nik^{F/F}$ and $Nik^{\Delta IE}$ assessed at the age of 3, 6 or 8 months. Images were taken at 10X magnification. n=3mice/group. **d** Gross image showing colon lymphoid follicles. Colon follicles were dissected using dissection scope and used for further analysis. **e** Western blot analysis of p52 in the small intestine (SI), Peyer's patches (PP), colon (col) and colon lymphoid follicles (Col LF) of WT mice. **f** Peyer's patch number and size assessed in 6-week old $Nik^{\Delta IE}$ mice. **g** H&E analysis of Peyer's patch from $Nik^{F/F}$ and $Nik^{\Delta IE}$ mice. Images were taken at 20X magnification. **h** Staining for M-cell marker UEA-1 in the Peyer's patches of $Nik^{\Delta IE}$ mice. Images were taken at 20X magnification. **i** Peyer's patch number and size assessed in 7-week old $Ikk\alpha^{\Delta IE}$ mice. **j** qPCR analysis for M-cell markers in the Peyer's patches of $Ikk\alpha^{\Delta IE}$ mice. **k** Fecal IgA measured in $Ikk\alpha^{\Delta IE}$ mice. **l** mRNA levels of *Nik* in $Nik^{F/F;VilERT2Cre}$ mice at 2-weeks after tamoxifen treatment. **m** Peyer's patch number and size assessed in $Nik^{F/F;VilERT2Cre}$ mice at 2-weeks after tamoxifen treatment. Results are expressed as mean ± SEM. Significance was determined using *t* test. $^*P < 0.05$; $^{**}P < 0.01$, $^{***}P < 0.001$.

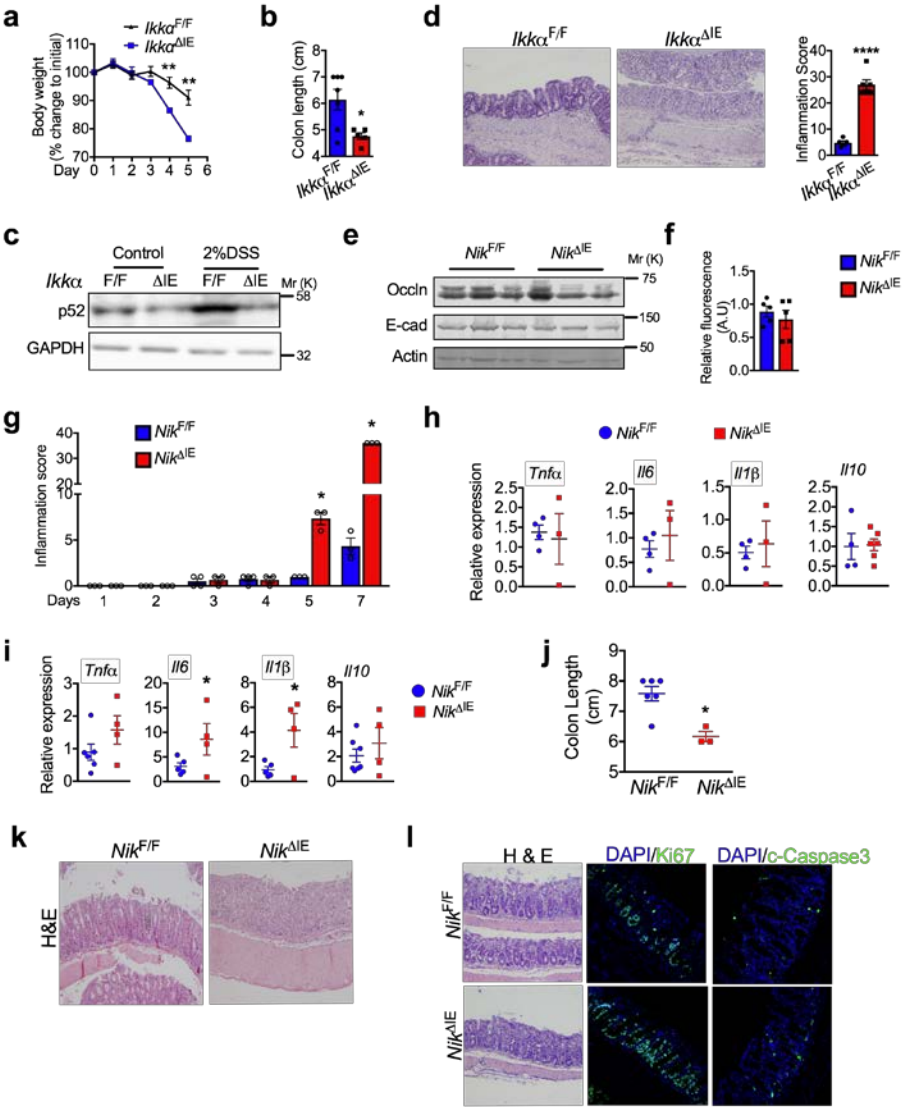

Supplementary Fig. 2

Loss of epithelial NIK-IKKα signaling increases colitis independent of pro-inflammatory changes. **a**-**d** Body weight (**a**), colon length (**b**), p52 expression (**c**) and H&E analysis of colon (**d**) in $Ikk\alpha^{F/F}$ and $Ikk\alpha^{\Delta IE}$ mice were treated with 2% DSS for 5-days. Images were taken at 10X magnification. n=4-7 mice/group. **e** Western blot analysis for occludin, e-cadherin in the colon of 6-week old $Nik^{\Delta IE}$ mice (n=3/group). **f** Intestinal permeability was assessed by FITC-dextran assay in 6-week old mice treated with 2% DSS for 7-days. **g** Inflammation score in $Nik^{\Delta IE}$ treated with 2% DSS for 0-7days **h**, **i** qPCR analysis of *Tnf-α*, *IL-6*, *IL-1β* and *Il10* in the colon epithelial cells of $Nik^{\Delta IE}$ treated with 2% DSS for 3-days (**h**) or 7-days (**i**). **j**, **k** Colon length (**j**) and colon inflammation (**k**) assessed by H&E at day-7 in $Nik^{F/F}$ and $Nik^{\Delta IE}$ mice following *Salmonella*-induced colitis. Images were taken at 10X magnification. **l** $Nik^{F/F}$ and $Nik^{\Delta IE}$ mice were lethally irradiated with 12Gy x-rays and 3-days post irradiation, the colon was assessed by H&E (for morphological and inflammatory changes), Ki67 (for cell proliferation) and cleaved Caspase3 (for apoptosis). Images were taken at 10X magnification. Results are expressed as mean ± SEM. Significance was determined using *t* test. *P < 0.05; **P < 0.01; ***P < 0.001, ****P < 0.0001.

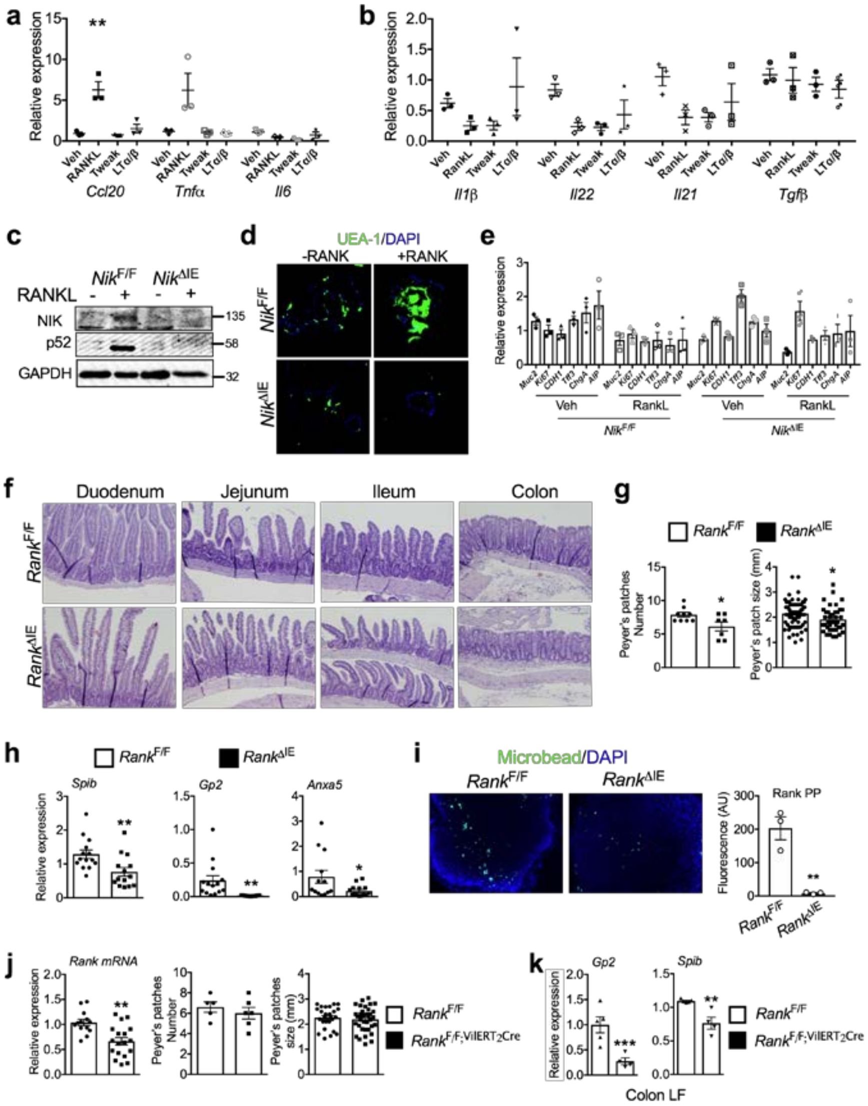

Supplementary Fig. 3

RANK is required for NIK-mediated M-cell maintenance. **a**, **b** qPCR analysis for cytokine expression in enteroids treated with Vehicle, 100ng/ml RANKL, 100ng/ml Tweak, 10ng/ml LTα/β for 72 hours. **c** Western blot analysis of NIK and p52 in lysates from duodenal enteroids that were treated with 100ng/ml RANKL for 72-hours. Experiment was done in triplicate. **d** UAE-1 lectin staining of duodenal enteroids that were treated with 100ng/ml RANKL for 72-hours. **e** qPCR analysis for epithelial and secretory markers in $Nik^{\Delta IE}$ enteroids treated with 100ng/ml RANKL for 72-hours. **f** H&E analysis of small intestine and colon of 6-week old $Rank^{\Delta IE}$ mice. Images were taken at 10X magnification. **g** Peyer's patch size and number assessed in $Rank^{\Delta IE}$ mice. **h** qPCR analysis of M-cell markers in the Peyer's patches of $Rank^{\Delta IE}$ mice. **i** Antigen sampling assessed using microbeads in the Peyer's patch of $Rank^{\Delta IE}$ mice. Images were taken at 20X magnification. **j** qPCR analysis for RANK in Peyer's patch, size and number of Peyer's patches in $Rank^{F/F;VilERT2Cre}$ mice at 2-weeks after tamoxifen treatment. **k** qPCR analysis of M-cell markers in the colon of $Rank^{F/F;VilERT2Cre}$ mice at 2-weeks after tamoxifen treatment. Results are expressed as mean ± SEM. Significance was determined using *t* test. *P < 0.05; **P < 0.01, ***P < 0.001.

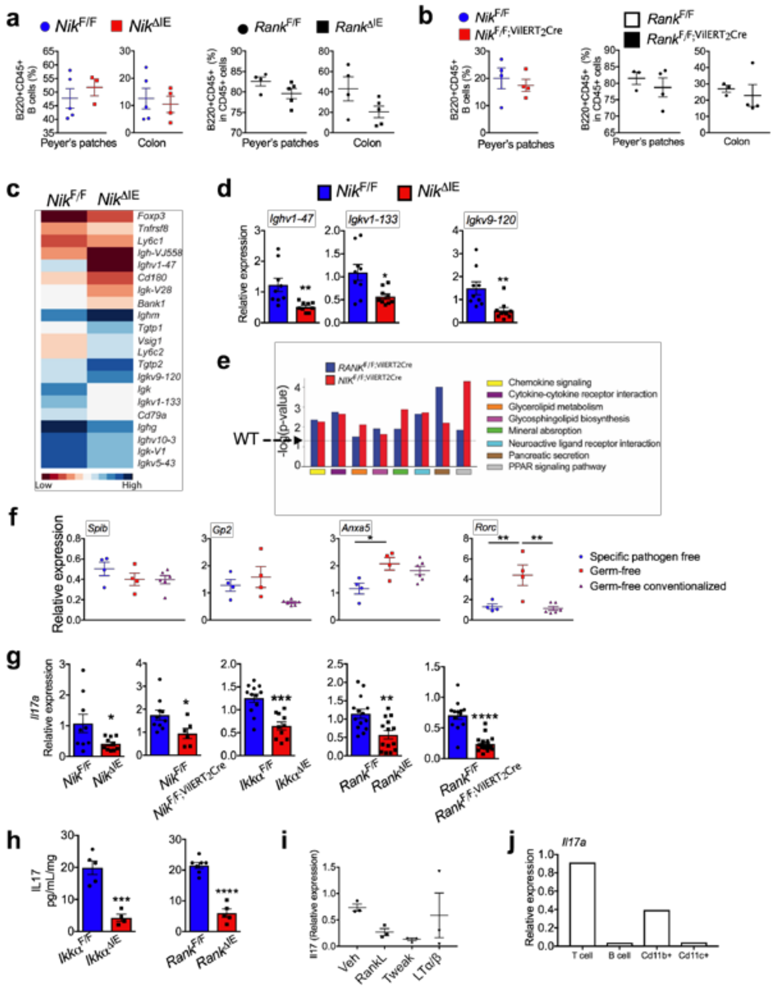

Supplementary Fig. 4

Loss of epithelial NIK decreases IL17 expression in PP T-cells. **a**, **b** Flow analysis for B-cells in Peyer's patches and colon of *Nik*$^{\Delta IE}$ and *Rank*$^{\Delta IE}$ mice (**a**) and *Nik*$^{F/F;VilERT2Cre}$ and *Rank*$^{F/F;VilERT2Cre}$ mice at 2-weeks after tamoxifen treatment (**b**). **c** Heat map of microarray analysis in the colon of 6-week old *Nik*$^{F/F}$ and *Nik*$^{\Delta IE}$ mice. n=3-4/group. **d** qPCR analysis of key genes involved in B-cell class switching assessed in the Peyer's patches of *Nik*$^{\Delta IE}$ mice; n=9 mice/group. **e** Pathway analysis of differentially expressed transcripts identified by RNA-seq in the Peyer's patches of *Nik*$^{F/F;VilERT2Cre}$ and *Rank*$^{F/F;VilERT2Cre}$ mice. The analysis was compared to WT controls which is normalized to 1. **f** qPCR analysis for the marker of M-cells and *Rorc* in the Peyer's patches of specific pathogen free (SPF), germ free (GF) and GF mice conventionalized for 2-weeks by fecal transplant from SPF donors. **g** qPCR analysis for *Il17a* expression in the peyers patches of mice with intestinal epithelial specific disruption of NIK, IKKα or RANK. n=6-14 mice/group. **h** IL17 protein levels assessed by ELISA in the colon of *Ikkα*$^{\Delta IE}$ and *RANK*$^{\Delta IE}$ mice that were treated with 2% DSS for 6-days. n=4-7 mice/group. **i** qPCR analysis for Il17 mRNA in the enteroids treated with100ng/ml RANKL, 100ng/ml Tweak, 10ng/ml LTα/β for 72-hours. Experiment was done in triplicate. **j** *Il17a* mRNA levels in the flow sorted-immune cells from the peyers patches of 10 wild type mice. Results are expressed as mean ± SEM.Significance was determined using *t* test or one-way ANOVA. *P < 0.05; **P < 0.01; ***P < 0.001; ****P < 0.0001.

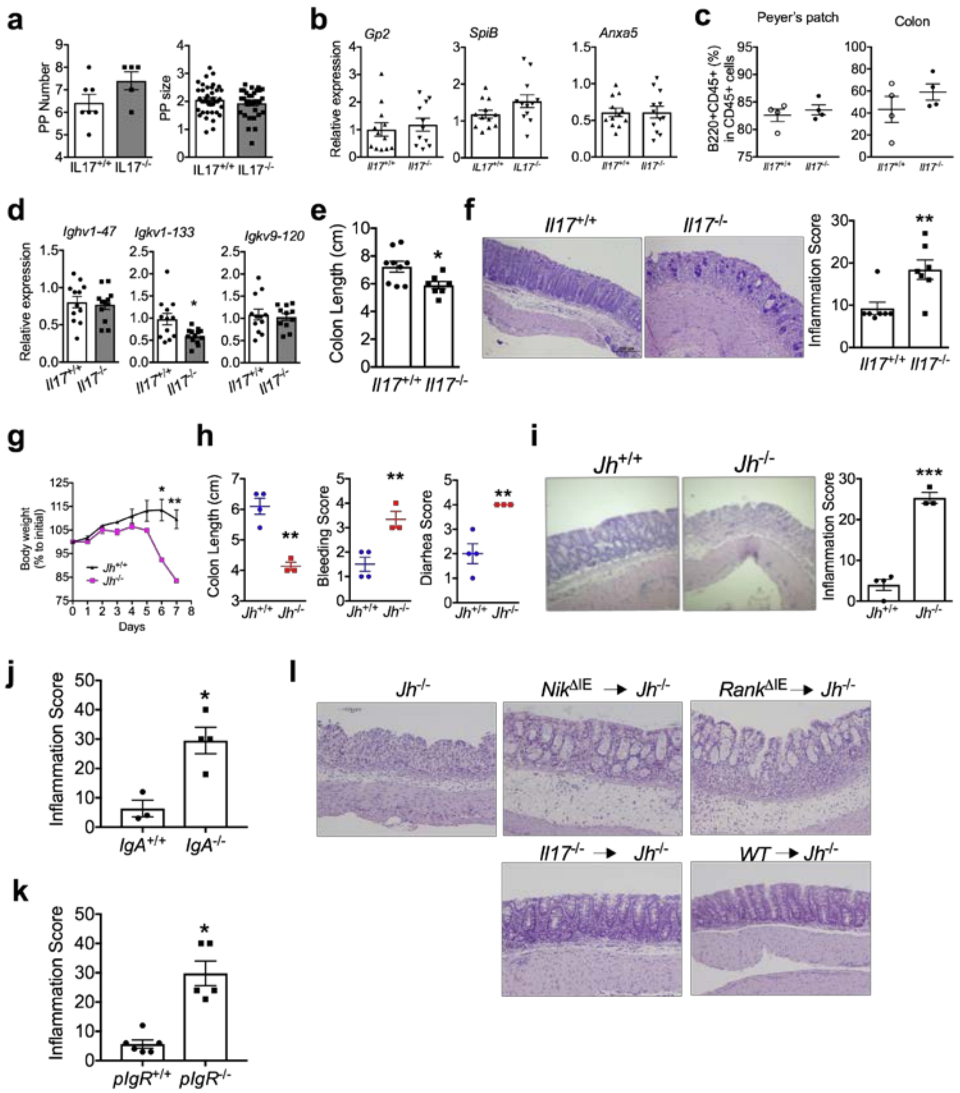

Supplementary Fig. 5

IL17-mediated IgA production is associated with protection against colitis. **a** Peyer's patch number and size assessed in *Il17*$^{-/-}$ mice. n=5-7 mice/group. **b** qPCR analysis for M-cell markers in the PP of *c* mice. **c** Flow analysis for B cells in the Peyer's patch and colon of *Il17*$^{-/-}$ mice. **d** qPCR analysis in the Peyer's patch of *Il17*$^{+/+}$ and *Il17*$^{-/-}$ mice. n=6 mice/group. **e**, **f** Colon length (**e**) and H&E analysis (**f**) in 8-week old *Il17*$^{-/-}$ mice treated with DSS for 7-days. Images were taken at 10X magnification. **g-i** Body weight (**g**), colon length, bleeding score and diarrhea score (**h**) and H&E analysis (**i**) of colon in 7-week old *Jh*$^{-/-}$ mice that were treated with 2% DSS for 7-days. Images were taken at 10X magnification. **j,k** Inflammation score in 6-week old *IgA*$^{+/+}$ and *IgA*$^{-/-}$ (**j**) and *pIgR*$^{+/+}$ and *pIgR*$^{-/-}$ mice (**k**) that were treated with 2% DSS for 7-days. **l** H&E analysis in 8-week old *Jh*$^{-/-}$ chimeras that were treated with DSS for 7-days. Images were taken at 10X magnification. Results are expressed as mean ± SEM. Significance was determined using *t* test. *P < 0.05; **P < 0.01; ***P < 0.001.

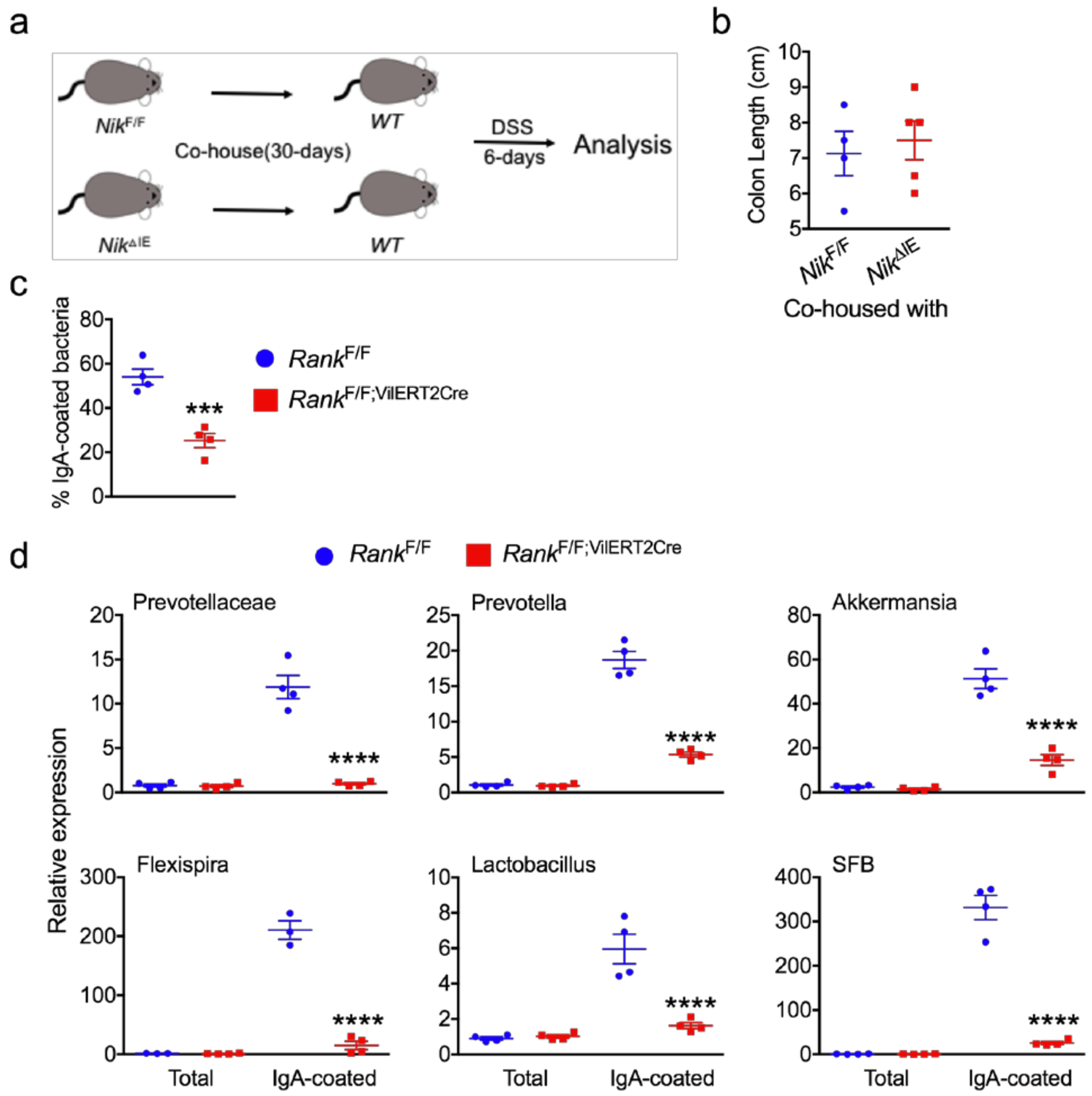

Supplementary Fig. 6

Epithelial RANK-NIK signaling is essential for IgA coating of colitogenic bacteria. **a** Schematic representation of co-housing of WT mice with $Nik^{F/F}$ or $Nik^{\Delta IE}$ for 30-days followed by DSS treatment. **b** Colon length of WT mice that were co-housed with either $Nik^{F/F}$ or $Nik^{\Delta IE}$ mice. **c** Flow analysis of feces sorted for IgA coated bacteria in $Rank^{F/F;VilERT2Cre}$ mice at 2-weeks after tamoxifen treatment. **d** qPCR analysis for bacterial DNA assessed in flow sorted IgA-coated bacteria in the feces of $Rank^{F/F;VilERT2Cre}$ mice. Results are expressed as mean ± SEM. Significance was determined using *t* test. ***P < 0.001; ****P < 0.0001.

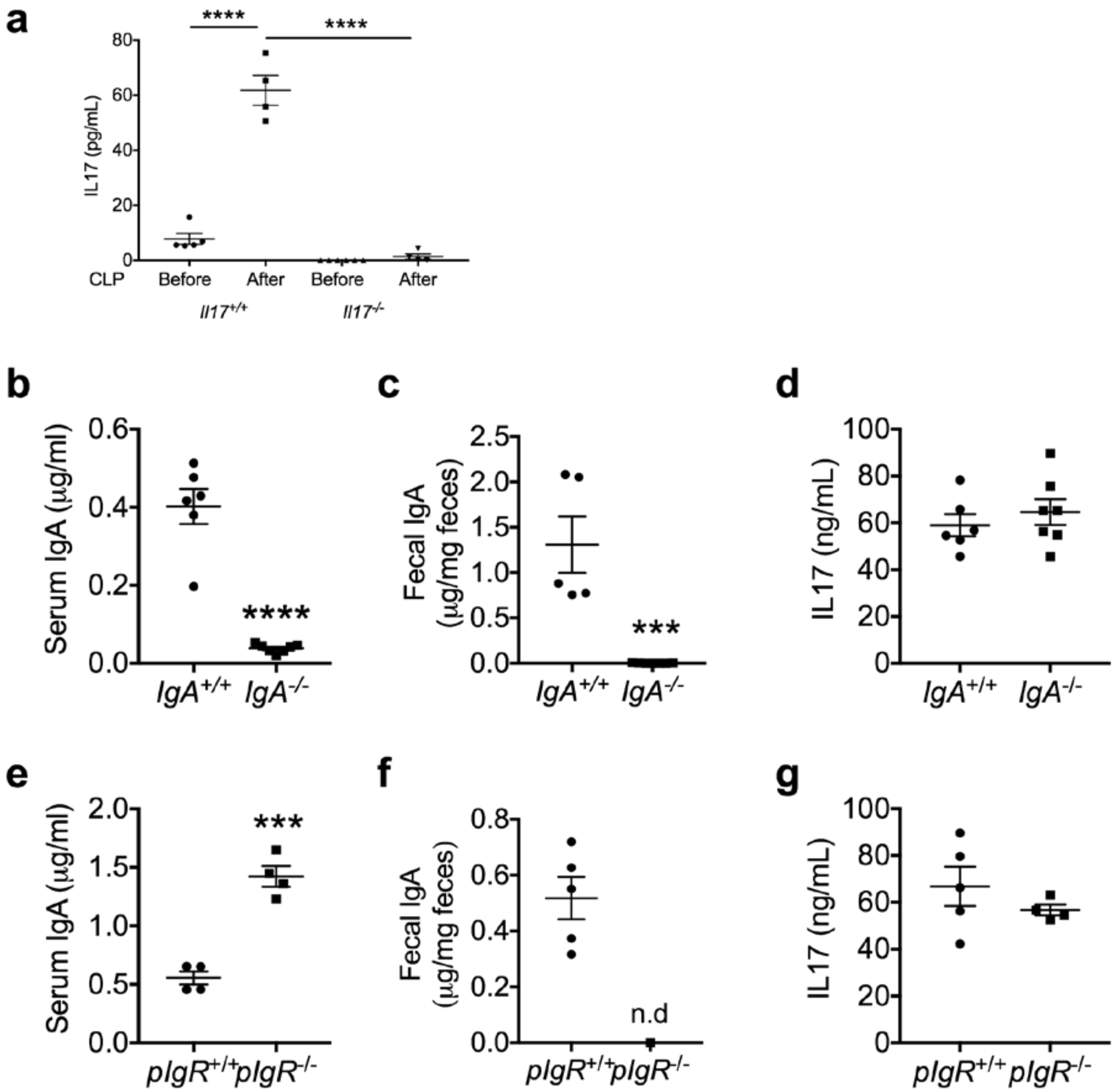

Supplementary Fig. 7

Serum IgA is protective against polymicrobial sepsis. **a** Serum IL17 at 6-hour post cecal ligation and puncture (CLP) in 6-week old *Il17*$^{-/-}$ mice following CLP. **b, c** Serum (**b**) and fecal IgA (**c**) assessed in *IgA*$^{-/-}$ mice. **d** Serum IL17 assessed at 6-hours post CLP in *IgA*$^{-/-}$ mice. **e, f** Serum (**e**) and fecal IgA (**f**) assessed in *pIgR*$^{-/-}$ mice. **g** Serum IL17 assessed in *pIgR*$^{-/-}$ mice at 6-hours post CLP. Results are expressed as mean ± SEM. Significance determined using *t* test or One-way ANOVA. ***P < 0.001; ****P < 0.0001.

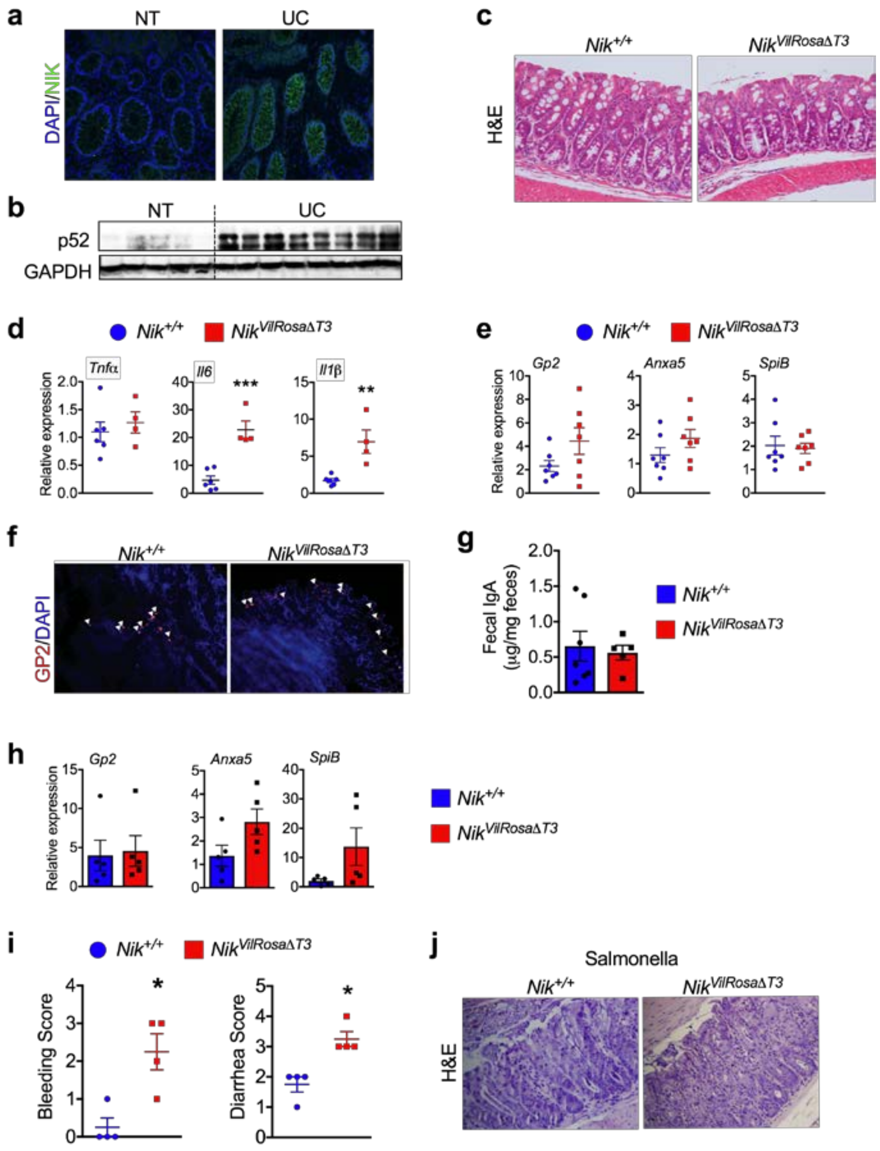

Supplementary Fig. 8

Constitutive activation of NIK increases proinflammatory response. **a, b** Expression of NIK and p52 assessed by immunostaining (**a**) and Western blot analysis (**b**) in ulcerative colitis (UC) and adjacent normal tissue (NT); n=5-8/group. **c** H&E analysis in untreated colon of $Nik^{+/+}$ and $Nik^{VRosa\Delta T3}$ mice. Images were taken at 10X magnification. **d** qPCR analysis of inflammatory genes in the colon of $Nik^{+/+}$ and $Nik^{VRosa\Delta T3}$ mice treated with 2% DSS. **e, f** qPCR analysis for M-cell markers in individual PP (**e**) or small intestine (**f**) from 6-week old $Nik^{+/+}$ and $Nik^{VRosa\Delta T3}$ mice. Images were taken at 20X magnification. **g** GP2 staining in the colon LF of 6-week old $Nik^{+/+}$ and $Nik^{VRosa\Delta T3}$ mice. **h** Fecal IgA measured in $Nik^{VRosa\Delta T3}$ mice. **i** Bleeding score and diarrhea score in $Nik^{+/+}$ and $Nik^{VRosa\Delta T3}$ mice treated with 2% DSS. **j** H&E analysis of colon of $Nik^{+/+}$ and $Nik^{VRosa\Delta T3}$ mice assessed 6-days after gavage with *Salmonella typi*. Images were taken at 10X magnification. Results are expressed as mean ± SEM. Significance was determined using *t* test. *P < 0.05; **P < 0.01; ***P < 0.001.

Supplementary Table 1. List of primers.

Primer name	Primer sequence
Nik F	TCCACAGAATGAAGGACAAGCA
Nik R	CACCTCGAGTCGTACCTTTTTGA
Tnf-α F	AGGGTCTGGGCCATAGAACT
Tnf-α R	CCACCACGCTCTTCTGTCTAC
Il-6 F	ACCAGAGGAAATTTTCAATAGGC
Tgfβ F	CAACCCAGGTCCTTCCTAAA
Tgfβ R	GGAGAGCCCTGGATACCAAC
Il-6 R	TGATGCACTTGCAGAAAACA
Il-1β F	AAGAGCTTCAGGCAGGCAGTATCA
Il-1β R	TGCAGCTGTCTAGGAACGTCA
Ikkα F	GGGTTATGCCAAAGATGTTGAT
Ikkα R	ACAGTGGCTGTGTACGG
Igkv9-120 F	CATGAGGGCTCCTGCACAGA
Igkv9-120 R	CAATGTCCTGACTTGCCCGA
Igkv1-133 F	TGATGAGTCCTGCCCAGTTCC
Igkv1-133 R	TGGTTGTCCAATGGTAACCGAC
Ighv1-47 F	GTCCTGCAAGGCTTCTGGCT
Ighv1-47 R	AATGTGGCCTTGCCCTTGAA
Gp2 F	GCTCAGTTGGCCTCTCAGAA
Gp2 R	CTGCTACCTCGAAGGGGACT
SpiB F	CAGCTGTCCAGGTCGTAGAAG
SpiB R	AACCACCATGCTTGCTCTG
Anxa5 F	CCGAAGGACTTCTGCATCA
Anxa5 R	GCTCTACTGCCTGCTCCAGT
Muc2 F	CCTGAAGACTGTCGTGCTGT
Muc2 R	GGGTAGGGTCACCTCCATCT
Alk Phos F	GTGCCAGAGAAAGAGAGAGA
Alk Phos R	TTTCAGGGCATTTTTCAAGGT
Chga F	GTCTCCAGACACTCAGGGCT
Chga R	ATGACAAAAGGGGACACCAA
Tff3 F	GCACCATACATTGGCTTGG
Tff3 R	AGAGCCCTCTGGCTAATGCT
Cdh1 F	AAAAGAAGGCTGTCCTTGGCC
Cdh1 R	GAGGTCTACACCTTCCCGGT
Ki67 F	AGCACAAAGAGACGGTCTAAGA
Ki67 R	CTCTGCCTCGTGACTGTGTT
Tnfrsf11a(*RANK*) F	CCGCTAGAGATGAACGTGGA
Tnfrsf11a(*RANK*) R	CTGATGAGAAGGGAGCCTCA
Foxp3 F	CTCGTCTGAAGGCAGAGTCA
Foxp3 R	TGGCAGAGAGGTATTGAGGG

Rorc F	TCCACTACGGGGTTATCACCT
Rorc R	AGTAGGCCACATTACACTGCT
Ahr F	CTCCTTCTTGCAAATCCTGC
Ahr R	GGCCAAGAGCTTCTTTGATG
Il17a F	TGAGCTTCCCAGATCACAGA
Il17a R	TCCAGAAGGCCCTCAGACTA
Il22 F	TCGCCTTGATCTCTCCACTC
Il22 R	GCTCAGCTCCTGTCACATCA
Il10 F	CTTACTGACTGGCATGAGGATCA
Il10 R	GCAGCTCTAGGAGCATGTGG
Prevotellaceae F	ATTGGAGGGCAAGTCTGGTG
Prevotellaceae R	CCGATCCCTAGTCGGCATAG
Prevotella F	CACGGTAAACGATGGATGCC
Prevotella R	GGTCGGGTTGCAGACC
Akkermansia F	CAGCACGTGAAGGTGGGGAC
Akkermansia R	CCTTGCGGTTGGCTTCAGAT
Flexispira F	AATACATGCAAGTCGAACGATGA
Flexispira R	AATCACCGTTTCCAGTGGCT
Lactobacillus F	TGGATGCCTTGGCACTAGGA
Lactobacillus R	AAATCTCCGGATCAAAGCTTACTTAT
SFB F	GACGCTGAGGCATGAGAGCA
SFB R	GACGGCACGGATTGTTATTC
