# Supplementary material for: Intestinal non-canonical NFκB signaling shapes the local and systemic immune response
Source: Nat Commun. 2019 Feb 8;10:660. doi: 10.1038/s41467-019-08581-8 (PMC6368617; doi:10.1038/s41467-019-08581-8)
Supplement: Supplementary file 3 — Description of Additional Supplementary Files [file 41467_2019_8581_MOESM3_ESM.pdf]

Description of Additional Supplementary Files

File Name: Supplementary Data 1
Description: RNA-SEQ data in the Peyer's Patch from *Nik*$^{F/F;VilERT2Cre}$ mice at 2-weeks after tamoxifen treatment.

File Name: Supplementary Data 2
Description: RNA-SEQ data in the Peyer's Patch from *Rank*$^{F/F;VilERT2Cre}$ mice at 2-weeks after tamoxifen treatment.

File Name: Supplementary Data 3
Description: List of common genes decreased (from RNA-SEQ) in the Peyer's Patch from *Nik*$^{F/F;VilERT2Cre}$ and *Rank*$^{F/F;VilERT2Cre}$ mice.

File Name: Supplementary Data 4
Description: List of common genes increased (from RNA-SEQ) in the Peyer's Patch from *Nik*$^{F/F;VilERT2Cre}$ and *Rank*$^{F/F;VilERT2Cre}$ mice.
