# Supplementary material for: Intestinal non-canonical NFκB signaling shapes the local and systemic immune response
Source: Nat Commun. 2019 Feb 8;10:660. doi: 10.1038/s41467-019-08581-8 (PMC6368617; doi:10.1038/s41467-019-08581-8)
Supplement: Supplementary file 8 — Reporting Summary [file 41467_2019_8581_MOESM8_ESM.pdf]

nature research

Corresponding author(s): Yatrik M Shah

Last updated by author(s): Jan 14, 2019

Reporting Summary

Nature Research wishes to improve the reproducibility of the work that we publish. This form provides structure for consistency and transparency in reporting. For further information on Nature Research policies, see Authors & Referees and the Editorial Policy Checklist.

Statistics

For all statistical analyses, confirm that the following items are present in the figure legend, table legend, main text, or Methods section.

n/a	Confirmed	
☐	☒	The exact sample size (n) for each experimental group/condition, given as a discrete number and unit of measurement
☐	☒	A statement on whether measurements were taken from distinct samples or whether the same sample was measured repeatedly
☐	☒	The statistical test(s) used AND whether they are one- or two-sided<br>*Only common tests should be described solely by name; describe more complex techniques in the Methods section.*
☒	☐	A description of all covariates tested
☒	☐	A description of any assumptions or corrections, such as tests of normality and adjustment for multiple comparisons
☐	☒	A full description of the statistical parameters including central tendency (e.g. means) or other basic estimates (e.g. regression coefficient) AND variation (e.g. standard deviation) or associated estimates of uncertainty (e.g. confidence intervals)
☐	☒	For null hypothesis testing, the test statistic (e.g. F, t, r) with confidence intervals, effect sizes, degrees of freedom and P value noted<br>*Give P values as exact values whenever suitable.*
☒	☐	For Bayesian analysis, information on the choice of priors and Markov chain Monte Carlo settings
☒	☐	For hierarchical and complex designs, identification of the appropriate level for tests and full reporting of outcomes
☒	☐	Estimates of effect sizes (e.g. Cohen's d, Pearson's r), indicating how they were calculated

Our web collection on statistics for biologists contains articles on many of the points above.

Software and code

Policy information about availability of computer code

Data collection	No commercial source or open source data was used in this study.
Data analysis	N/A

For manuscripts utilizing custom algorithms or software that are central to the research but not yet described in published literature, software must be made available to editors/reviewers. We strongly encourage code deposition in a community repository (e.g. GitHub). See the Nature Research guidelines for submitting code & software for further information.

Data

Policy information about availability of data

All manuscripts must include a data availability statement. This statement should provide the following information, where applicable:

- Accession codes, unique identifiers, or web links for publicly available datasets
- A list of figures that have associated raw data
- A description of any restrictions on data availability

The microarray data have been deposited in GEO database under the accession number GSE86194 [https://www.ncbi.nlm.nih.gov/geo/query/acc.cgi?acc=GSE86194]. The 16S sequencing data have been deposited in Sequence Read Archive (SRA) database under the accession numbers SAMN10753948-SAMN10753962 [https://www.ncbi.nlm.nih.gov/sra/?term=SAMN10753948-SAMN10753962]. The RNA data have been deposited in SRA database under the accession number SRP109133 [https://www.ncbi.nlm.nih.gov/sra/?term=SRP109133]. The source data underlying Fig 4a and Supplementary Fig 4e and uncropped blots are provided as a Source Data File.

Field-specific reporting

Please select the one below that is the best fit for your research. If you are not sure, read the appropriate sections before making your selection.

☒ Life sciences ☐ Behavioural & social sciences ☐ Ecological, evolutionary & environmental sciences

For a reference copy of the document with all sections, see nature.com/documents/nr-reporting-summary-flat.pdf

Life sciences study design

All studies must disclose on these points even when the disclosure is negative.

Sample size	Sample size for animal experiments were determined based on our previous experience. For experiments involving enteroids, the experiments were done in triplicated and repeated at least twice.
Data exclusions	No data were excluded from the analysis
Replication	There were no findings that were not replicated.
Randomization	For our experiments no randomization was applied.
Blinding	Investigators were not blinded to group allocation or data analysis. H&E scoring was done in a blinded fashion by the pathologist.

Reporting for specific materials, systems and methods

We require information from authors about some types of materials, experimental systems and methods used in many studies. Here, indicate whether each material, system or method listed is relevant to your study. If you are not sure if a list item applies to your research, read the appropriate section before selecting a response.

Materials & experimental systems

n/a	Involved in the study	
☐	☒	Antibodies
☒	☐	Eukaryotic cell lines
☒	☐	Palaeontology
☐	☒	Animals and other organisms
☐	☒	Human research participants
☒	☐	Clinical data

Methods

n/a	Involved in the study	
☒	☐	ChIP-seq
☐	☒	Flow cytometry
☒	☐	MRI-based neuroimaging

Antibodies

Antibodies used	NIK (Catalog # 4994, Cell signaling technology, Danvers, MA), p52 (Catalog # 4882, Cell signaling technology), E-cadherin (Catalog # 3195, Cell signaling technology), Occludin (Catalog # 13409-1-AP, Proteintech, Rosemont, IL), Actin (Catalog # 66009-1-Ig, Proteintech Inc) and GAPDH (Catalog # sc-25778, Santa Cruz Biotechnology Inc). HRP-conjugated or licor secondary antibodies (Catalog #7074, #7076, #5470, #5151 from Cell signaling technology). APC eFluor780-conjugated anti-CD45 (cat #47-0451-82), PE-Cy7-conjugated anti-CD4 (cat#25-0041-82), APC-conjugated anti-Foxp3 (cat#17-5773-82), FITC-conjugated anti-CD3e (cat#11-0031-81), PE-conjugated anti-CXCR5 (cat#12-7185-80), and eFluor450-conjugated anti-PD-1 (cat#48-9981-80) from eBioscience (San Diego, CA) or Alexa Fluor647-conjugated anti-B220 (cat#103229) from BioLegend (San Diego, CA), PE-conjugated anti-IL17A (cat#506903) (BioLegend, San Diego, CA)
Validation	The validation results for all comercial antibodies used are available on the manufacturers website. Our study with the knockouts with expected decrease in the expression of the genes tested further validates the

Animals and other organisms

Policy information about studies involving animals; ARRIVE guidelines recommended for reporting animal research

Laboratory animals	Mouse, C57BL6, Males, 5-16 weeks.
Wild animals	Study did not involve wild animals
Field-collected samples	Study did not involve samples collected from field
Ethics oversight	Animal studies were approved by the IACUC committee at the University of Michigan.

Note that full information on the approval of the study protocol must also be provided in the manuscript.

Human research participants

Policy information about studies involving human research participants

Population characteristics	Deidentified biopsies from IBD patients were collected.
Recruitment	Deidentified biopsies from IBD patients were collected.
Ethics oversight	University of Michigan IRB committee

Note that full information on the approval of the study protocol must also be provided in the manuscript.

Flow Cytometry

Plots

Confirm that:

☒ The axis labels state the marker and fluorochrome used (e.g. CD4-FITC).

☒ The axis scales are clearly visible. Include numbers along axes only for bottom left plot of group (a 'group' is an analysis of identical markers).

☒ All plots are contour plots with outliers or pseudocolor plots.

☒ A numerical value for number of cells or percentage (with statistics) is provided.

Methodology

Sample preparation	For intracellular staining , cells were fixed and permeabilized with 1X permeabilization buffer (eBioscience) following cell surface staining according to the manufacturer's protocol. All surface antibodies were used with 1:100 dilution and the intracellular staining antibodies were 1:200 dilution.
Instrument	BD LSRFORTESSA X-20 (BD Biosciences)<br>MoFlo Astrios I (Beckman Coulter, IN)
Software	FlowJo 10.2 software (FlowJo LLC, Ashland, OR)
Cell population abundance	Purity was confirmed based on a stringent gating strategy.
Gating strategy	For the gating strategy, CD4 T cells were gated on single cells, CD45+CD3+CD4+cells, the subsets were then further gated with Foxp3+ as T-reg cells, GFP+ (RORC(γt)-EGFP) as Th17 cells, PD1+CXCR5+ as T-follicular cells. B cells were gated with single cells, then CD45+B220+ cells. Gating was done on cells for APC-eflour 780-CD45, FITC-CD3, and PEcy7-CD4 positivity (CD45+CD3+CD4 +).

☐ Tick this box to confirm that a figure exemplifying the gating strategy is provided in the Supplementary Information.
